# Supplementary material for: Lutzomyia longipalpis Antimicrobial Peptides: Differential Expression during Development and Potential Involvement in Vector Interaction with Microbiota and Leishmania
Source: Microorganisms. 2021 Jun 11;9(6):1271. doi: 10.3390/microorganisms9061271 (PMC8230673; doi:10.3390/microorganisms9061271)
Supplement: Supplementary file 1 [file microorganisms-09-01271-s001.zip › microorganisms-1073982-supplementary.pdf]

Supplementary Materials: The following are available online at www.mdpi.com/xxx/s1.

Table S1: Primers

Reference	Name	Sequence
GenBank: KP030755	dsAtt-F	TAATACGACTCACTATAGGGAGACATCTATTGGTGGTGGGCTC [1]
	dsAtt-R	TAATACGACTCACTATAGGGAGAGCGATGAGAAAGACCAAGT [1]
GenBank: KP030758	dsDef2-F	TAATACGACTCACTATAGGGAGAGTTGTTGTTGTTGTTGGAGT [1]
	dsDef2-R	TAATACGACTCACTATAGGGAGAAATCCATGTCCGATGCAA [1]
Adapted from Molina-Cruz *et al*. 2008	dsLacZ-F	TAATACGACTCACTATAGGGAGATATCCGCTCACAATTCCACA [1]
	dsLacZ-R	TAATACGACTCACTATAGGGAGAGAGTCAGTGAGCGAGGAAGC [1]
Tinoco-Nunes *et al*. 2016	rtAtt-F	ATGGGCATGGCAGCGTCTCT
	rtAtt-R	AGGCTGATCCTCTGGGTCCTGT
Tinoco-Nunes *et al*. 2016	rtCec-F	TGGCAGTCCTGACCACTGGA
	rtCec-R	CTTCTCCACTGAACGGTGAACG
GenBank: EF491251	rtDef1-F	GCTGCAAATCCTGCAAAGA
	rtDef1-R	CCCAAGGAGGTCACAGGTTA
Tinoco-Nunes *et al*. 2016	rtDef2-F	ATCCATCCTTTATGCAACCG
	rtDef2-R	GCCTTTGAGTCGCAGTATCC
GenBank: MW269862	rtDef3-F	GACTCTTTGCGATTGGAGGA
	rtDef3-R	ATCCACCACTGAAGCCCTTA
GenBank: MW269863	rtDef4-F	TGAAGAGATTCCTGAAGCACC
	rtDef4-R	CCACCCTTATGTCCAAGAGC
Meireles-Filho *et al*. 2008	rtRP49-F	GACCGATATGCCAAGCTAAAGCA
	rtRP49-R	GGGGAGCATGTGGCGTGTCTT
Di-Blasi *et al*. 2015	LeishActin-F	GTCGTCGATAAAGCCGAAGGTGGTT
	LeishActin-R	TTGGGCCAGACTCGTCGTACTCGCT
Yang *et al*. 2002	Bac16S-F (P891F)	TGGAGCATGTGGTTTAATTCGA
	Bac16S-R (P1033R)	TGCGGGACTTAACCCAACA

[1] Underlined nucleotides correspond to T7 adaptor for RNA polymerase binding.

Table S2: Similarities shared between *L. longipalpis* defensin amino acid sequences

	Def2	Def4	Def1	Def3
Def2	n/a [1]	40.82 %	39.18 %	30.61 %
Def4	40.82 %	n/a [1]	53.93 %	36.67 %
Def1	39.18 %	53.93 %	n/a [1]	70.45 %
Def3	30.61 %	36.67 %	70.45 %	n/a [1]

[1] not applicable

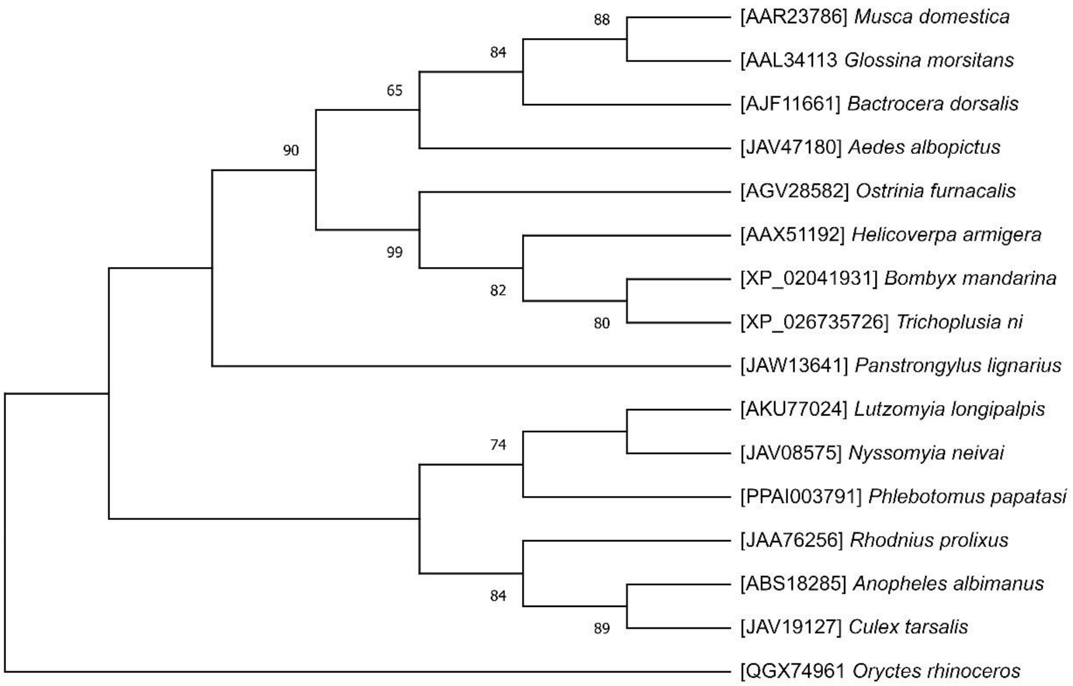

Figure S1: Phylogram of attacin amino acid sequences: Phylogram was constructed with *L. longipalpis* and other insects attacin sequences inferred by Maximum Likelihood method. Numbers on branch nodes indicate bootstrap values higher than 50%. Branch lines are followed by GenBank or Vector Base accession number and species names.

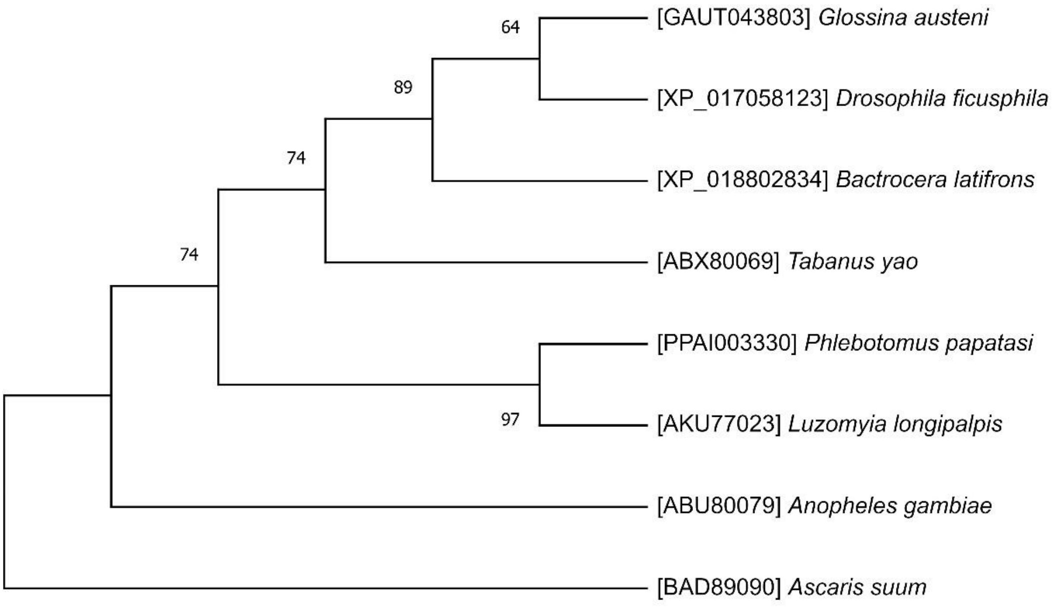

Figure S2: Phylogram of *L. longipalpis* cecropin amino acid sequence: Phylogram was constructed with *L. longipalpis* cecropin and other closely related insect sequences inferred by Maximum Likelihood method. Numbers on branch nodes indicate bootstrap values higher than 50%. Branch lines are followed by GenBank or Vector Base accession number and species names.

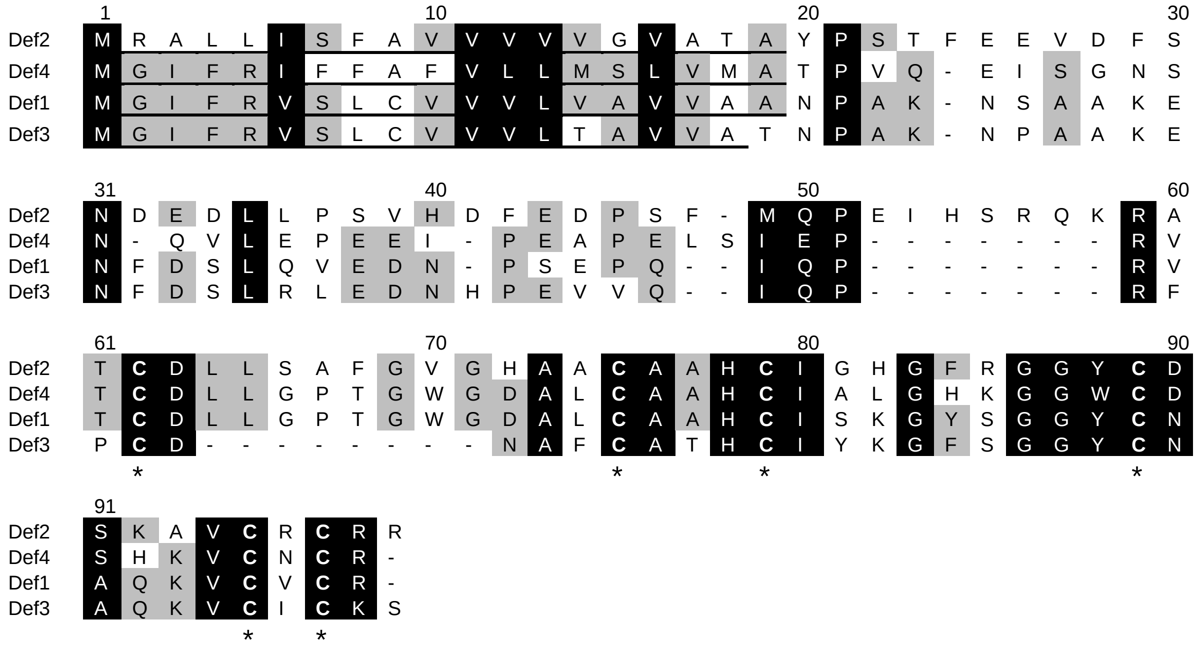

Figure S3: Amino acid sequences of *L. longipalpis* defensins: Name of sequences are indicated on the left side and position of residues are indicated by numbers above the sequences. Predicted signal peptide regions are underlined. Residues sharing higher similarity are shaded in black and lower similarity in grey. Six conserved cysteine residues from defensin family are indicated by asterisks.

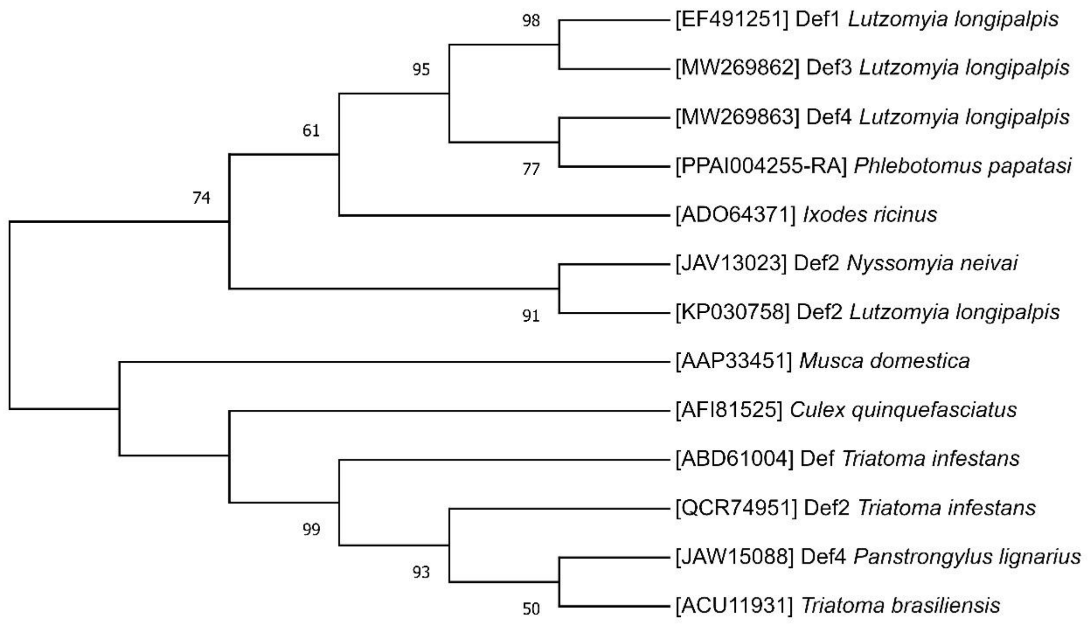

Figure S4: Phylogram of defensin amino acid sequences: Phylogram was constructed with *L. longipalpis* and other insects defensin sequences inferred by Maximum Likelihood method. Numbers on branch nodes indicate bootstrap values equal or higher than 50%. Branch lines are followed by GenBank or Vector Base accession number, defensin name (when more than one defensin sequence was identified in a single species), and species names.

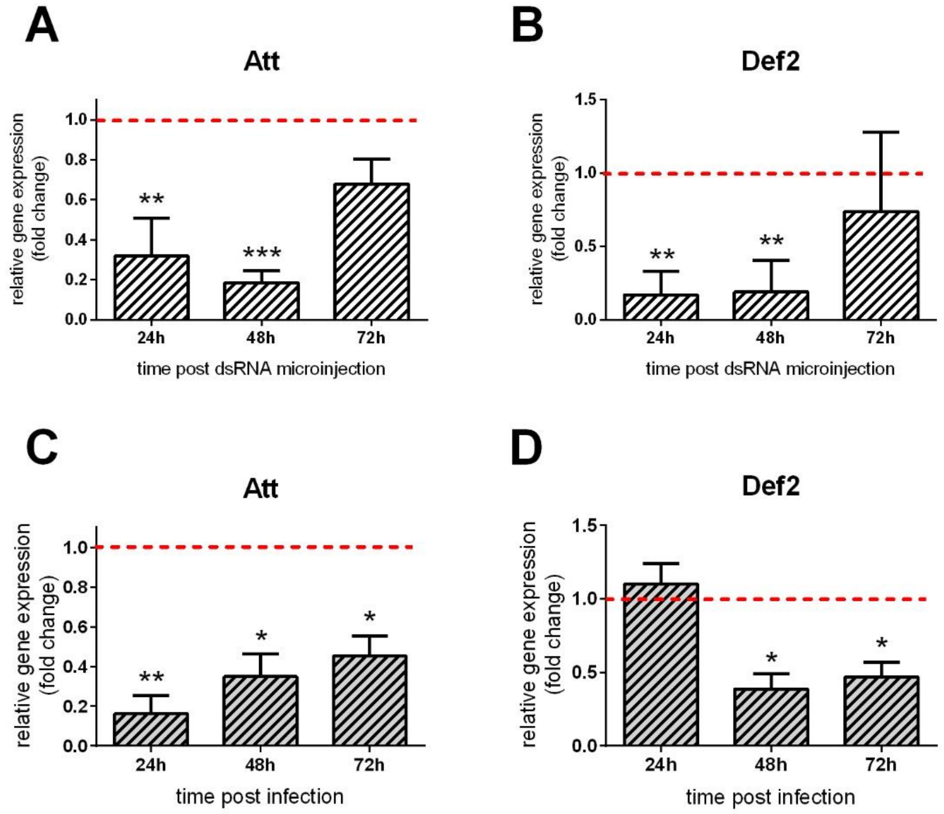

Figure S5: Relative gene expression of AMPs in dsRNA injected female sand flies. (**A** and **B**) gene expression in dsRNA injected and fed on sucrose. (**C** and **D**) gene expression in dsRNA injected and infected with *Leishmania*. (**A** and **C**) attacin expression in Att-dsRNA injected; (**B** and **D**) defensin 2 expression in Def2-dsRNA injected. Y-axis represents relative gene expression as fold change values in comparison to the control group injected with LacZ dsRNA. X-axis represents time post dsRNA microinjection in insect thorax (**A** and **B**), or post infection (**C** and **D**). Vertical bars represent the average values of three independent experiments, and error bars represent the standard error. Two-way ANOVA test were carried for significant differences (* $p < 0.05$; ** $p < 0.01$; *** $p < 0.001$).
